# Time-Lapse Imaging of the Dynamics of CNS Glial-Axonal Interactions *In Vitro* and *Ex Vivo*

**DOI:** 10.1371/journal.pone.0030775

**Published:** 2012-01-27

**Authors:** Kalliopi Ioannidou, Kurt I. Anderson, David Strachan, Julia M. Edgar, Susan C. Barnett

**Affiliations:** 1 Institute of Infection, Immunity and Inflammation, University of Glasgow, Glasgow, United Kingdom; 2 Institute of Cancer Sciences, The Beatson Institute for Cancer Research, Glasgow, United Kingdom; Université Pierre et Marie Curie-Paris6, INSERM, CNRS, France

## Abstract

**Background:**

Myelination is an exquisite and dynamic example of heterologous cell-cell interaction, which consists of the concentric wrapping of multiple layers of oligodendrocyte membrane around neuronal axons. Understanding the mechanism by which oligodendrocytes ensheath axons may bring us closer to designing strategies to promote remyelination in demyelinating diseases. The main aim of this study was to follow glial-axonal interactions over time both *in vitro* and *ex vivo* to visualize the various stages of myelination.

**Methodology/Principal Findings:**

We took two approaches to follow myelination over time: i) time-lapse imaging of mixed CNS myelinating cultures generated from mouse spinal cord to which exogenous GFP-labelled murine cells were added, and ii) *ex vivo* imaging of the spinal cord of *shiverer* (*Mbp* mutant) mice, transplanted with GFP-labelled murine neurospheres. We demonstrate that oligodendrocyte-axonal interactions are dynamic events with continuous retraction and extension of oligodendroglial processes. Using cytoplasmic and membrane-GFP labelled cells to examine different components of the myelin-like sheath, we provide evidence from time-lapse fluorescence microscopy and confocal microscopy that the oligodendrocytes' cytoplasm-filled processes initially spiral around the axon in a corkscrew-like manner. This is followed subsequently by focal expansion of the corkscrew process to form short cuffs, which then extend longitudinally along the axons. We predict from this model that these spiral cuffs must extend over each other first before extending to form internodes of myelin.

**Conclusion:**

These experiments show the feasibility of visualizing the dynamics of glial-axonal interaction during myelination over time. Moreover, these approaches complement each other with the *in vitro* approach allowing visualization of an entire internodal length of myelin and the *ex vivo* approach validating the *in vitro* data.

## Introduction

Myelination is a fundamental biological process in the developing vertebrate nervous system. The myelin sheath is formed by the spiral wrapping of the oligodendrocyte's plasma membrane extensions around the axon [1], [2]. Myelin sheaths foster rapid and efficient conduction of electrical impulses along axons. The development of the myelin sheath, in which the processes of oligodendrocytes in the central nervous system (CNS) wrap around axons to form multilamellar insulating layers, has allowed for the evolution of highly complex but compact nervous systems [3]. However, the specialised process of wrapping and compaction of the myelin sheath is not well understood [4].

Myelin contains an array of proteins [5]; myelin basic protein (MBP) and the proteolipid proteins (PLP/DM20) being the two major CNS myelin proteins. During the active phase of myelination, each oligodendrocyte must produce as much as approximately 5–50×10^3^ µm^2^ of myelin membrane surface area per day [6]. Before final maturation and myelin formation, oligodendrocytes go through many stages of development which have been characterised by a panel of antigenic markers, by their morphological phenotypes and their mitotic and migratory properties [7], [8].

Electron microscopy studies [2] and time-lapse imaging of green fluorescent protein (GFP) labelled oligodendrocytes *in vivo* [9], [10] and *in vitro* [11], [12] have helped to elucidate the mechanisms of glial-axonal interaction during the initial stages of myelination. Nonetheless, the processes of axonal ensheathment and myelin compaction are still not fully understood. Two models have dominated over the past few years. First, it has been proposed that the leading edge of the myelin sheath, which aligns along the axon in a sheet-like manner, forms an initial wrap, which then moves underneath the growing sheet to form the next wrap [4] and described as a “carpet crawler” model [12]. In the second model, a narrow oligodendrocyte cell process spirals around the axon and when a sufficient number of wraps have been generated by turns around the axon, the spirals extend laterally into overlapping sheets [13], described as the “serpent” model in [12]. It has also been suggested that these two models are not mutually exclusive and it is likely that intermediate mechanisms might be involved [4], [13].

In this study we used both morphological analysis of fixed tissue and time-lapse imaging of cytoplasmic or membrane-located GFP in oligodendroglia, to examine the early stages of myelination *in vitro* and *ex vivo*. Our studies suggest that the oligodendrocyte process, both during myelination *in vitro* and after transplantation *ex vivo*, initially wraps in a corkscrew-like spiral around the axon and that this is followed by a spreading of the membrane to form short cuffs of glial cytoplasm, prior to sheath extension and the formation of internodes of myelin. These studies demonstrate the feasibility of visualizing the dynamic glial-axonal interactions that occur during myelination over time.

## Methods

### Animals

The following mice were used and maintained in Glasgow University Veterinary Research Facility: homozygous *shiverer* mice (*shi/shi*) on the C3H/101 genetic background, wild type mice on the C57BL/6 (Charles River Laboratories International Inc, Ormiston, Scotland) background, mice expressing enhanced green florescent protein (GFP) under the *β-actin* promoter [14] on the C57BL/6 genetic background (C57BL/6-Tg(ACTB-EGFP)1Osb/J) and mice expressing cyan fluorescent protein (CFP) under the *Thy1* promoter. The *β-actin* promoter drives cytoplasmic expression of GFP in all cells. S*hiverer* is an autosomal recessive myelin basic protein *(Mbp)* mutant with deficiency of myelin sheath formation in the CNS [15]. The *Thy1-CFP* line [16] (B6.Cg-Tg(Thy1-CFP)23Jrs/J), which was originally supplied as a double transgenic, expressing GFP under the S100 promoter, was kindly provided by Professor Wesley Thompson. For some experiments the *Thy1-CFP* line was crossed with *shiverer* mice to produce Thy1-CFP*shvi/shi mice which had both labelled axons and the *Mbp* mutation. All experimental mice were bred at the facility. Mice had access to food and water, *ad libitum*. Furthermore, all procedures were carried out in accordance with the guidelines, set forth by the Animals Scientific Procedures Act, under a project license (No. 6003895) granted by the UK Home Office and with the approval of the University of Glasgow Ethical Review Process Applications Panel.

### Isolation and culture of GFP and wild type neurospheres

Neurospheres were generated from the striatum of *β*-actin GFP-transgenic mice, expressing cytoplasmic GFP (cyto-GFP) or wild type mice as previously described [17]. Neurospheres were cultured in the presence of 20 ng/mL mouse epidermal growth factor (EGF, Peprotech, London, UK) and passaged weekly for expansion, by mechanical dissociation in the same neurosphere medium. The cultures were incubated at 37°C in a humidified atmosphere of 7% CO_2_/93% air, changing two thirds of the media every 2–3 days. GFP neurospheres were used in both the *in vitro* and *in vivo* experiments. In some experiments the neurospheres were labelled with farnesylated GFP (farns-GFP) by lentivral transduction (lentivrus gift from Prof J Verhaagen, Netherlands Institute for Neuroscience, NIN). Triturated neurospheres were incubated with 10 µl of 2.2×10^9^ tu/ml of the viral supernatant overnight and maintained in neurosphere medium.

### Myelinating cultures

The method for generating myelinating cultures is based on previously published work [17], [18]. Wild-type, or *shiverer (shi/shi)* female mice were time-mated, with the day of plugging denoted as embryonic day 0.5 (E0.5), and embryos were collected on embryonic day 13.5 (E13.5). The spinal cord was dissected, dissociated mechanically and enzymatically (0.25% trypsin, Invitrogen, Paisley UK; 1.0% collagenase, ICN Pharmaceuticals, Basingstoke, UK). Enzymatic activity was stopped by the addition of (0.52 mg/mL soyabean trypsin inhibitor, 3.0 mg/mL bovine serum albumin, and 0.04 mg/mL DNase, Sigma-Aldrich, Poole, Dorset UK). Cells were triturated through a glass pipette and spun at 800 rpm for 5 min and the pellet resuspended in 5 ml of plating medium (PM; 50% DMEM, 25% horse serum, 25% Hanks balanced salt solution without Ca^2+^ and Mg^2+^, and 2 mM L-glutamine, Invitrogen). The dissociated spinal cord cells were plated initially onto coverslips supporting a monolayer of astrocytes, or in most cases on top of PLL- coated coverslips at a density of 150,000 cells/100 µl and were then placed in a 35-mm Petri dish. The cells were left to attach for 2–3 hours in the incubator, after which 300 µl of PM and 500 µl of differentiation medium was added [17], [18] which contained DMEM (4,500 mg/mL glucose, 10 ng/ml biotin, 0.5% hormone mixture (1 mg/mL apotransferrin, 20 mM putrescine, 4 µM progesterone, and 6 µM selenium (formulation based on N2 mix [19]), 50 nM hydrocortisone, and 0.5 mg/ml insulin (all reagents were from Sigma). Cultures were maintained by replacing half of the medium with fresh medium three times a week. After 12 days in culture, insulin was excluded from the differentiation media. The cultures were maintained for up to 28 days in a humidified atmosphere of 7% CO_2_/93% air at 37°C. For time-lapse imaging ascorbic acid (0.5 µl/1 ml, Sigma) was added to the medium for enhancement of cell survival.

For the study of glial axonal interactions under time-lapse microscopy, we used two modifications to the cultures: i) Cyto-GFP labelled neurospheres generated from the beta-actin mouse or farns-GFP labelled neurospheres generated from WT mice and infected with a lentivirus encoding farnesylated GFP (which labels cellular membranes) were added to WT or *shiverer* myelinating cultures or ii) myelinating cultures generated from wild type and/or beta actin-GFP mice (to give a ratio of green and non-green cells) with exogenously added wild type neurospheres which were infected with a lentivirus containing farnesylated GFP or the lentivirus dsRedexpress2-IRIS-GFP (gift Prof J Verhaagen) which labels the cytoplasm both red and green. S*hiverer* mice were used in these experiments for several reasons; they harbour a mutation in the *Mbp1* gene [20], [21] leading to truncation and degradation of the MBP protein [22], [23] and fail to synthesise compact CNS myelin [24]. Since MBP is not detectable by immunohistochemistry in *shiverer* [22], its presence in cultures or after transplantation (see below) can be ascribed to exogenous cells, in this case, added neurospheres. Previously using the electron microscope we demonstrated that compact myelin formed in these cultures [25], however in some of the time-lapse imaging in the current study we may only be imaging a single oligodendroglial wrap. For this reason we have termed the myelin as myelin-like although it is highly likely we are following myelin sheath formation. In a similar vein when following the cell types during time lapse we can only assume cell lineage based on classic morphology and have therefore termed the cells as oligodendroglia-like for this reason.

### Transplantation of spinal cord neurospheres

Neonatal *shiverer* mice 19–21 days of age were recipients of neurosphere transplantation, as described previously [26]. Briefly, the mice were anesthetised by inhalation of 5% isofluorane which was reduced to ∼2% during surgery, in combination with a mixture of nitrous oxide and oxygen (0.3 l/min O_2_/0.7 l/min N_2_O). Rimadyl–Carprofen (Pfizer Animal Health, Tadworth, Surrey UK) was administered subcutaneously at the start of surgery, for pain relief. A short vertical incision was made with a scalpel blade over the thoraco-lumbar region of the spine. With the aid of an operating microscope, the transverse laminae of a vertebra were broken (laminectomy) to reveal the spinal cord. A small opening was made in the dura with a sterile needle and a cell suspension of cyto-GFP or farns-GFP neurospheres was injected using a CellTram Oil manual micromanipulator (Eppendorf Ltd, Cambridge, UK) at a rate of 1 µl/min, using a glass microelectrode that was inserted into the exposed spinal cord. One injection was made into the dorsal spinal cord, avoiding the midline dorsal vein. A total volume of 3–5 µl of cell suspension containing, approximately 5×10^4^ cells/µl was injected, at the depth of <1 mm. The electrode was left in place for an additional 2 min to minimise back-flow of cells. No immunosuppressant treatment was used. Mice were sacrificed at various time points 3 days, 7–10 days, 14 days and 4 weeks (±3 days) after transplantation.

### Tissue processing and immunohistochemistry

Animals were euthanised with CO_2_ and perfused transcardially with saline followed by 4% paraformaldehyde. In some cases, the spinal cords were dissected out and placed in 0.1 M glycine in phosphate buffered saline (PBS), then cryo-protected in 20% sucrose overnight at 4°C. The spinal cords were embedded in OCT and frozen rapidly in isopentane, cooled in liquid nitrogen. The rostro-caudal segment at the lumbar thoracic junction of the spinal cord encompassing the transplant site was embedded and sectioned dorso-ventrally. Cryostat serial sections (10 µm) were cut, mounted onto 3-aminopropyltiethoxysilane (APES, Sigma) coated slides and stored at −20°C. Before immunostaining, sections were air dried at room temperature (RT) for 10–20 min and then rehydrated in PBS for 10 min. The sections were permeabilised with methanol at −20°C for 10 min and blocked with 10% normal goat serum in PBS, for 1 hour at RT, followed by incubation in primary antibody in the same blocking solution overnight at 4°C. Antibodies used were: rabbit anti-GFP (1∶1000 Abcam, Cambridge, UK), mouse anti-GFP (1∶250, Abcam) for GFP transplanted cells, rat anti-MBP (1∶500, Serotec, AbD Serotec Oxford, UK) for mature oligodendrocytes, rabbit anti-Caspr (1∶1000; gift from Dr Elior Peles) for paranodal regions, mouse anti-GFAP (1∶1000, Sigma) for astrocytes and mouse anti-phosphorylated neurofilament (SMI-31) or mouse anti- non phosphorylated neurofilament (SMI-32) (1∶1500 Affiniti Research Products Ltd, Exeter, UK) for axons. The slides were washed three times with PBS and then incubated with appropriate fluorescent-conjugated secondary antibodies (Cambridge Biosciences) for 1 hour at RT. The slides were washed three times with PBS and then mounted in Citifluor antifade (Citifluor Ltd, London, UK) mounting medium.

### Immunohistochemistry and antibodies for myelinating cultures

Neurites/axons were visualized using a monoclonal antibody against phosphorylated neurofilament (SMI-31, IgG1, 1∶1500) or non phosphorylated neurofilament (SMI-32, IgG1, 1∶1500). Myelin or myelin-like sheaths (PLP/DM20) were detected using the AA3 antibody directed against PLP/DM20 (1∶100, anti-rat, hybridoma supernatant, [27], gift Dr Pfeiffer, University of Connecticut) or MBP (1∶500 for cells, anti-rat, Serotec). Morphological changes and the differentiation of oligodendrocytes in spinal cord myelinating cultures were visualized using the O4 antibody (IgM, hybridoma supernatant, [28]. Rabbit anti-GFP (1∶1000, Abcam) or mouse anti-GFP (1∶250, Abcam) were used to detect GFP labelled cells.

For cell surface labelling, primary antibodies (diluted in DMEM) were applied for 1 hour at RT. The cultures were washed in DMEM followed by PBS before fixing in warm (37°C) 4% paraformaldehyde for 20 min. After washing in PBS the appropriate secondary fluorescent-conjugated antibody (diluted in DMEM) was added for 1 hour at RT. For co-labelling with intracellular antigens, cells were subsequently washed in PBS, permeabilized with methanol at −20°C, for 10 min and blocked with 10% normal goat serum in PBS for 1 hour at RT. Primary antibodies, diluted in 10% normal goat serum, were incubated on the coverslips overnight at 4°C. The following day the coverslips were washed three times in PBS at RT and the appropriate fluorochrome-conjugated secondary antibodies were added for 1 hour at RT. The coverslips were washed and subsequently mounted in Vectashield (Vector Laboratories Ltd, Peterborough, UK).

### Maintenance of the explants for *ex vivo* imaging

The protocol was based on studies by Kerschensteiner and colleagues [29]. To keep the spinal cord explants viable during the 3–5 hour imaging, it is vital to ensure sufficient O_2_ and nutrient supply as well as a constant pH and temperature. Therefore all procedures were performed in a combination of F12+L-glutamine, CO_2_ independent medium and DMEM+Glutamax (4 g/L D-glucose) or Neurobasal A medium (Invitrogen) that had been bubbled with 95% O_2_ and 5% CO_2_ for at least 15 min before imaging. During dissection, the temperature was kept low by placing the mouse on aluminium foil with ice underneath, to protect the tissue from hypoxia. During imaging the explant was superfused with pre-warmed O_2_-bubbled medium. The temperature of the explant was maintained at 35–37°C using a heating stage, super fused with pre-warmed medium. Generally the tissue was kept well oxygenated after careful dissection and steady flow rates and temperature were established to create conditions as close to physiological as possible and to avoid drift during recording.

### Imaging of oligodendrocytes

For quantification of myelin sheaths we used our previously described methods [17]. For detailed morphological analysis of axons, oligodendrocytes and myelin sheath formation, images were captured by laser scanning confocal microscopy using either an Olympus FV1000 or Zeiss 710 confocal microscope. Image processing and movies were made with FV10 ASW (Olympus, Essex, UK) Full Version Viewer software. Complex interactions between structures were analysed from maximum intensity projections in the z dimension and visualised with Volocity (Version 5) and Imaris imaging software (Version 7.1.1) which provided high resolution volume rendering of multichannel 3D data sets. Further image processing was performed using Image J 1.44 and Fiji-win 32 software. Manual tracking using Fiji-win 32 was used to follow the dynamics of cyto-GFP and farns-GFP cell movements in time-lapse imaging..

### Multiphoton microscopy

Multiphoton microscopy was performed using a LaVision BioTec 2-photon TRIM scope or a Zeiss 7MP. The LaVision system consisted of a Nikon Eclipse TE2000 inverted stand, Olympus long working distance 20× 0.95 NA water immersion objective, and Coherent Chameleon II laser tuned to 830 nm. Fluorescence was detected using non-descanned detectors (NDD, Hamamatsu H6780-01-LV 1 M for <500 nm detection and H6780-20-LV 1 M for >500 nm detection). A dichroic filter (Chroma 475 DCXR) was used to separate spectrally the second harmonic signal, when present, from the GFP emission of the transplanted cells. Band pass filters (Semrock 435/40 and Chroma 525/50) were used to further filter the emission for the SHG and GFP channels respectively. The Zeiss system consisted of an Axio Imager upright stand, 20×, 1.0 NA water immersion objective, and Chameleon II laser. To keep tissue stationary during *ex vivo* imaging it was glued, dorsal part side up to a plastic cover slip with cyanoacrylate (Vetbond, 3 M Health Care Ltd, Leicestershire, UK). The cover slip was cut to fit in a perfusion chamber (Harvard Apparatus ltd, Kent, UK) where it was held in place by grease. The perfusate was equilibrated with oxygen and thermostated to 35–37°C. The chamber was placed on the motorized stage of the upright microscope and fluorescent cells located using epifluorescence with (blue) excitation. The wavelength of the laser was set to either 840 nm (to excite predominantly CFP) or 940 nm (to excite predominantly GFP). Detection channels selected light with wavelength <485 nm (for CFP) and 500–550 nm (for GFP).

### Time-lapse microscopy

The Nikon time-lapse microscope TE2000 is fitted with a Nikon perfect focus system (PFS) to maintain focus over the imaging. PFS requires cells plated on 35 mm Petri dish containing a 14 mm glass microwell (MatTek Corporation MA, USA). The system has a temperature-controlled 37°C chamber, provided with an oxygen supply and images were acquired using a 40× short distance 0.75 NA air objective. Analysis was performed with MetaMorph (Version 5) imaging software, which compensate for stage shift, vibration or similar small whole field movement that can occur during time-lapse acquisition.

## Results

### Characteristics of mouse myelinating cultures

Previously, we found a supporting astrocyte monolayer was necessary for cell survival and myelination in rat myelinating cultures [17], [18]. Although the presence of the astrocyte monolayer is somewhat more efficient for myelination, the optical properties for imaging would be improved by not imaging through an astrocyte monolayer. We therefore compared the effect of a mouse or rat astrocyte monolayer to support the mouse myelinating cultures compared to a PLL coated substrate. Neurite density was similar on all substrates tested (around 70% of SMI-31-immunoreactivity per field of view) but there was a small but significant increase in myelination in mouse cultures plated on rat or mouse neurosphere derived astrocytes, compared to cultures plated on PLL alone (means were 29±0.7% standard error myelinated fibres on PLL compared to 42±0.9% standard error and 44±0.6% standard error on mouse and rat astrocytes respectively, p<0.05; data not shown). As myelination was relatively high even in the absence of a supporting astrocyte substrate, we carried our subsequent studies on myelinating cultures plated on PLL alone.

**Figure 1:**
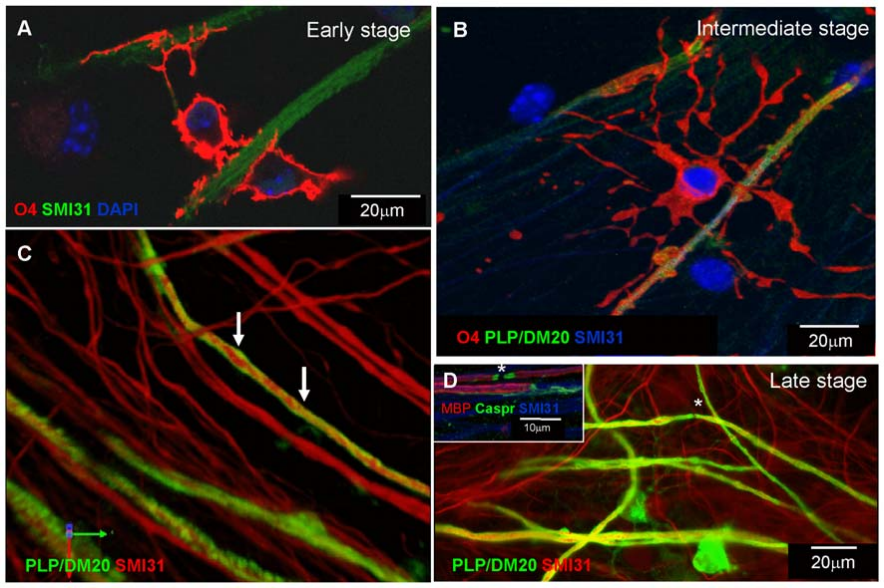
Static images of myelination from mouse cultures *in vitro* using differentiation markers. **A–B**) Confocal image acquired using FV1000 with 60× 0.75NA (A–C) showing the initial contact between oligodendrocytes (O4, red) and neurites (SMI-31, green) in a wild type mouse myelinating culture. After DIV 9 the oligodendrocyte's processes appear to align along neurites **B**). Subsequent stages in axon/oligodendrocyte contact and wrapping where PLP/DM20 can be detected alongside expression of the O4 antibody. The staining suggests that myelin sheaths wrap around segments of the axon at this stage (solid regions of red and green; arrows) around DIV 9. **C**) Image of a culture after 28 days *in vitro*. The oligodendrocyte's many processes appear to “fill-out” (solid green sheaths) from the initial spirals (arrows) of the membrane visualised by PLP/DM20 (green) around SMI-31 (red) neurites. **D**) Single contiguous myelin sheaths begin to appear around 17–18 days *in vitro* using anti-PLP/DM20. Nodes of Ranvier are apparent between the internodes of myelin (asterisk) using an antibody to the nodal protein Caspr (insert). Image taken with and a Zeiss Axioplan II, 20×, 0.75NA (D) and similar images were observed from 5–10 separate experiments.

**Figure 2:**
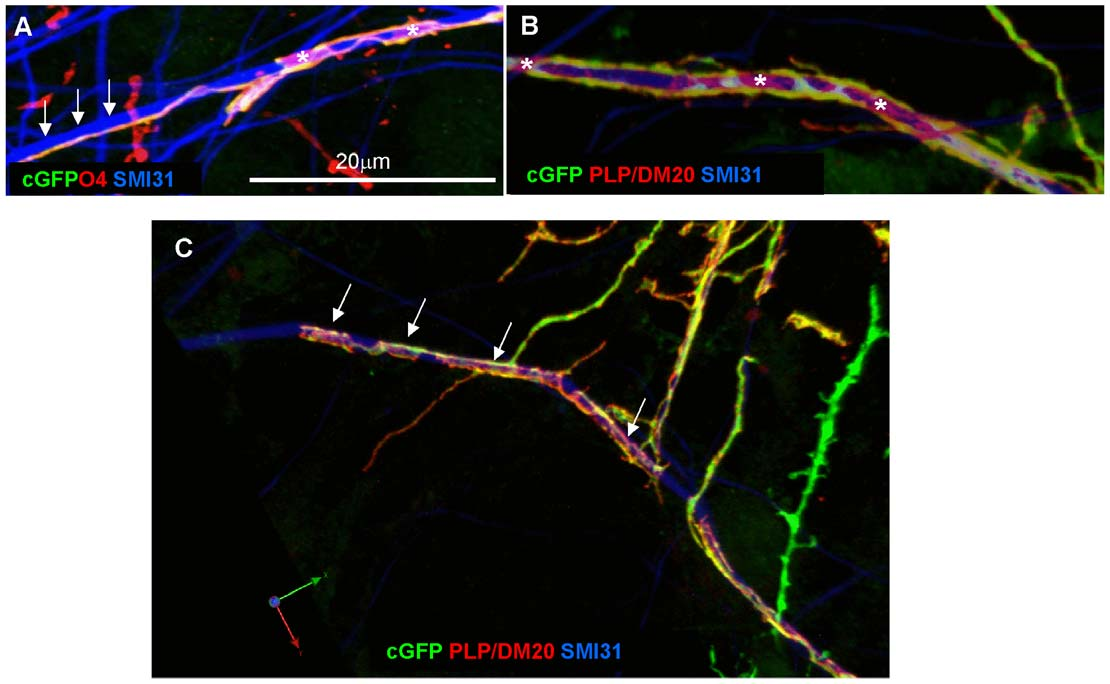
Cyto-GFP labelled neurospheres generate oligodendrocytes which spiral cell process around neuritis. **A**) Neurospheres expressing cyto-GFP under the *β-actin* promoter were added on day 6 to myelinating cultures prepared from wild type mouse embryos. Confocal imaging (Zeiss 710, 63×, 1.4NA) on DIV 21 demonstrates that cell processes initially form spirals around neurites. Since cyto-GFP (green) is only present in the cytoplasm we co-labelled with the O4 antibody which labels myelin membrane to determine the membrane and cyto-GFP are contiguous. It can be seen that at some point the GFP and O4 immunoreactivity are contiguous over the neurite (SMI-31, blue, arrowheads). **B**) Staining with anti-PLP/DM20 (red) and anti-GFP shows that myelin membrane lacking cyto-GFP extends from the cyto-GFP strands. **C**) Many cytoplasmic cuffs of cyto-GFP labelled with anti-PLP/DM20 can be seen along the neurite (arrows, blue). Representative images from at least 3 separate experiments.

As observed for rat myelinating cultures, there was a temporal progression in glial-axonal interactions in the mouse cultures that culminated in the formation of compact myelin sheaths, as demonstrated previously by electron microscopy [25]. As we planned to examine glial-axonal interaction in these cultures using time-lapse microscopy, we first characterised the stages of myelination, using immunocytochemistry with cell differentiation specific markers in wild type cultures (Fig. 1). We then confirmed that exogenous GFP-labelled neurospheres differentiated in the same manner by co-labelling wild type cultures with the anti-GFP and differentiation markers (Fig. 2). Fig. 1A shows the initial contact between an oligodendrocyte (O4, red) and neurites SMI-31 (green). The oligodendrocyte's processes appeared to align with neurites, then prior to the formation of a myelin-like sheath (Fig. 1C), the membranous processes “filled-out” over the axon from the initial narrow spirals (Fig. 1B, C). Later stages of myelination were defined by an increased intensity of PLP/DM20 or MBP staining (Fig. 1D) compared to intermediate stages (Fig. 1C) and localisation of axonal Caspr to the lateral edges of adjacent MBP or PLP/DM20 stained internodes (Fig. 1D inset).

Cyto-GFP labelled neurospheres were added to wild type myelinating cultures to generate a proportion of oligodendrocytes that expressed the *cyto-GFP* reporter gene (Fig. 2). At early stages of myelination we observed co-expression of the antigen recognised by the O4 antibody (membrane marker) and cyto-GFP-positive ribbon-like spirals of cytoplasm extending along and around axons (Fig. 2A; arrows and arrowheads). Unlike cyto-GFP which was confined to this narrow, ribbon-like structure, intense expression of sulphatides (as identified by the O4 antibody) was sometimes observed as sheath-like immunoreactivity along the nerve fibre (asterisk Fig. 2A). A similar pattern was observed for PLP/DM20, a transmembrane protein that is carried by vesicular transport through the biosynthetic pathway to myelin [30], [31] (Fig. 2B, asterisk). PLP/DM20 was also observed in the gaps between the cyto-GFP “ribbons”, presumably representing deposition in the adjacent plasma membrane (asterisk Fig. 2B). In Fig. 2C extension of the myelin like-sheath over an axon is visualised as short cuffs where PLP/DM20 appears to spread out from the cyto-GFP spirals.

### *In vitro* time-lapse imaging of OPC-like cells in *shiverer* myelinating cultures

**Figure 3:**
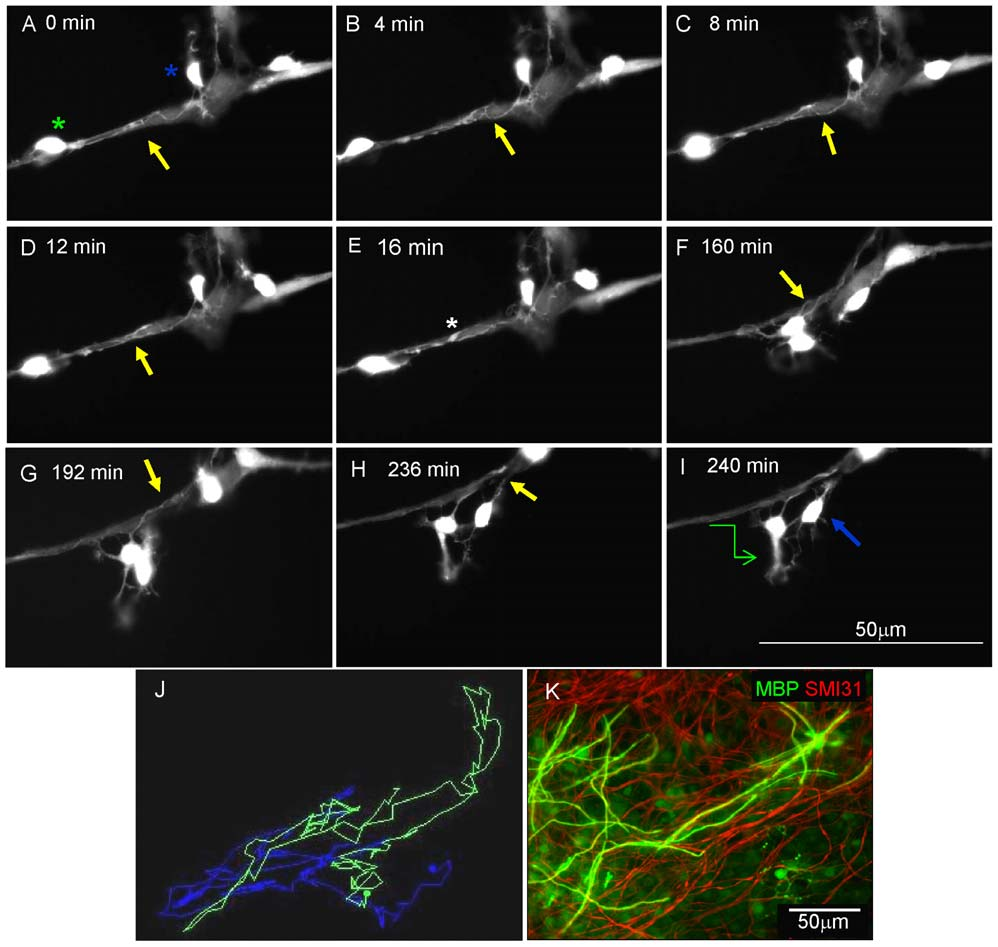
*In vitro* time-lapse imaging of cyto-GFP labelled OPC-like cells in *shiverer* myelinating cultures reveal dynamic cellular interactions. Neurospheres expressing cyto-GFP under the *β-actin* promoter were added to myelinating cultures prepared from *shiverer* embryos on DIV 13 and visualised on DIV 17 over 24 hours in 4 min intervals, using a Nikon TE2000 time-lapse microscope (40×, 0.75NA short distance working lens) with perfect focus. **A–I**) Images captured from a time-lapse sequence illustrate cells with morphology typical of oligodendrocyte progenitor cells (OPCs). Spirals of processes appear over the neurites. Both the cell soma and processes are highly motile, continually moving along the neurites. Processes appear to move over the nerve bundles (arrows and asterisk). **J**) Manual tracking of the cell bodies of the entire video illustrates the pathway of the putative OPC-like cells (asterisks green and blue) over time, demonstrating their highly motile behaviour. **K**) A sister culture was immunostained for myelin (PLP/DM20; green) and neurites/axons (SMI-31; red) on DIV 27, demonstrating that the added cyto-GFP neurospheres myelinated the axons. See Video S1. Representative images from at least 2 separate experiments.

Having assessed glial-axonal interactions in static images *in vitro*, we visualised the progression between these stages over time using time-lapse microscopy. S*hiverer* cultures to which cyto-GFP labelled neurospheres were added 4 days previously were imaged after 17 days in vitro (DIV) every 4 min for 24 hours (Video S1). Cyto-GFP positive cells with OPC-like morphology actively migrated along neuritic fascicles. Thin processes emanating from their cell bodies appeared to spiral around neurites (Fig. 3A–I, illustrates a 4 hour section of the video) and formed occasional small, and transient, thickenings (asterisk Fig. 3E). Process extension was highly dynamic and the pattern of association with the neurites changed continually. Manual tracking of the cell bodies during the complete video illustrates the pathway of the OPC-like cells (asterisks green and blue) over time, demonstrating their highly motile behaviour (Fig. 3J). A sister myelinating culture was immunolabelled for MBP (myelin) and neurites (SMI-31) after 28 days to illustrate and confirm the formation of myelin-like sheaths (Fig. 3K).

### Time-lapse imaging of fluorescently labelled oligodendrocytes in association with neurites *in vitro*

**Figure 4:**
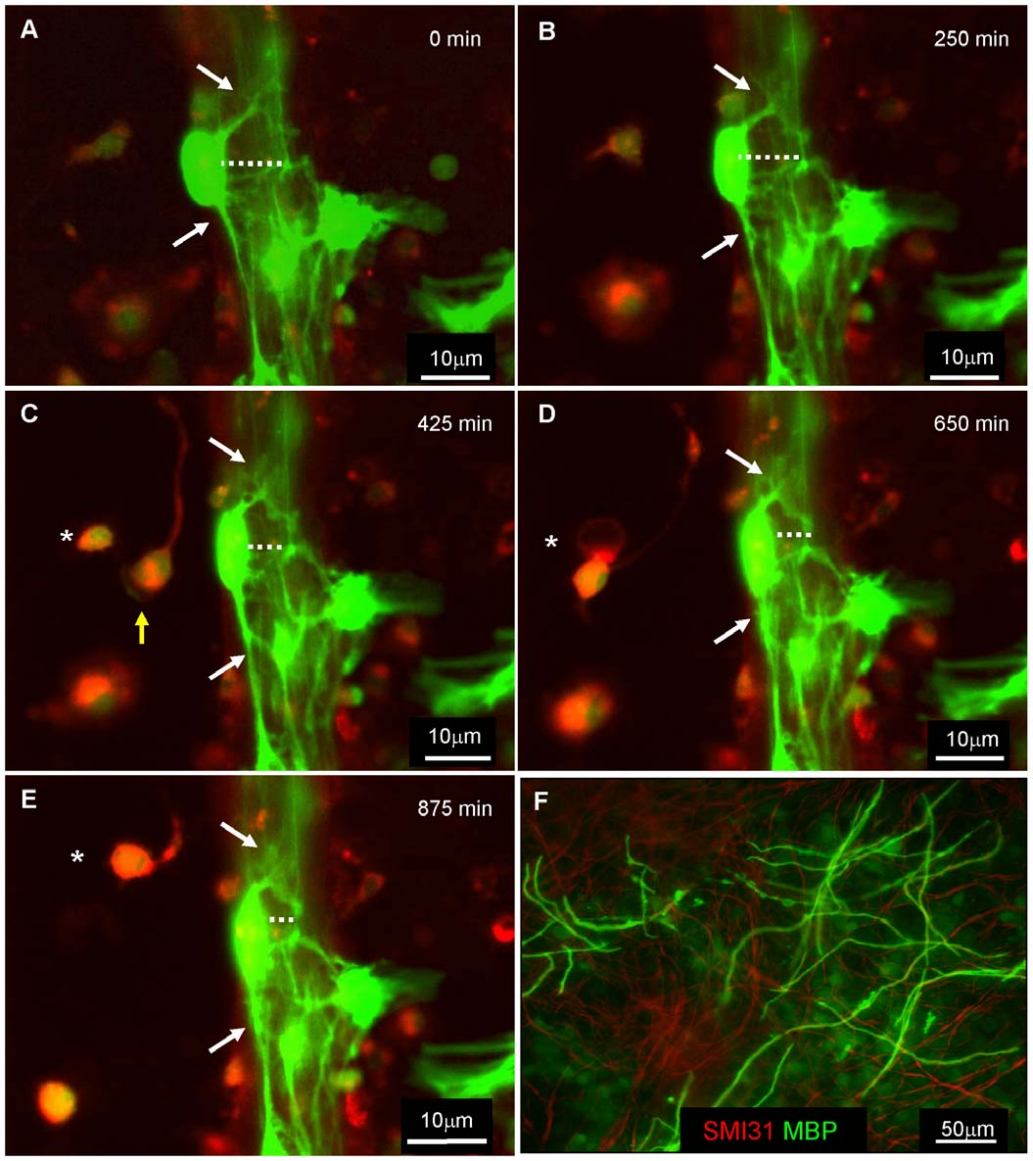
Time-lapse imaging of fluorescently labelled cells in association with neurites *in vitro*. **A–E**) Myelinating cultures generated from a mix of wild type/beta-actin mice were visualised using time-lapse microscopy (Nikon TE2000 (60×, 0.75NA) over 16 hr in 5 min intervals on DIV 27 after the addition of wild type neurospheres previously infected with lentivirus carrying dsRed/GFP gene and addition of cyto-GFP cells. Two cell types were followed over time, one that expressed DS red/cyto-GFP and the other cyto-GFP. **A–E**) Strongly positive green cells typical of cyto-GFP morphologically resembled oligodendrocytes in contact with neurite bundles. The membrane appears to ruffle and form flaps/bubbles (arrow). In addition, the soma changes its location with respect to the neurite processes, over time, by moving closer to the neurite bundle. **C–E**) Dynamic imaging over 7.5 hours of a dsred/GFP labelled cell (asterisk) which was engulfed by a cell resembling a microglial cell (yellow arrow). This fluorescence was very much weaker than the cells generated from the beta-actin cyto-GFP mouse. Time frames obtained with 40× magnification (long distance working lens) and without perfect focus. See Video S2. **F**) Immunostaining of a similar culture around the same time confirmed MBP expression. Representative video from 1 experiment, seen in duplicate.

Myelinating cultures were occasionally prepared from a mix of wild type/β-actin GFP mice with exogenously added cyto-GFP neurospheres (which differentiate into oligodendrocytes and astrocytes), and incubated with the lentivirus encoding dsRedexpress2-IRIS-GFP and visualised on DIV 27. Both lentivirus and cyto-GFP cells were added to this particular culture since the GFP label from lentivirus carrying the IRIS-GFP dual fluorophore was very weak. This experiment allowed us to visualise several cellular interactions at once. The dynamic nature of the cultures can be seen in representative stills (Fig. 4A–E) and video (Video S2) in which a red microglial cell (yellow arrow) engulfs a dying cell (*). A cyto-GFP labelled oligodendrocyte soma seems to to move laterally (in relation to the orientation of the image) towards neighbouring neurites (dotted line). However, it is also possible that the movement may represent the rotation of the entire nerve fascicle. Additionally there are dynamic changes in the processes of the cyto-GFP positive cell (arrows). The GFP labelled process appears to thicken with the formation of membrane protrusions. These images are suggestive of the oligodendrocyte process extending membrane and cytoplasmic material along the presumptive myelin sheath (supplemental video 2). This time-lapse image was taken at stages of differentiation, when myelin-like sheath formation is dominant, as observed in a culture stained around the same time with anti-MBP (Fig. 4F).

### Confirmation of corkscrew-like spiralling of oligodendroglial processes in fixed cultures

**Figure 5:**
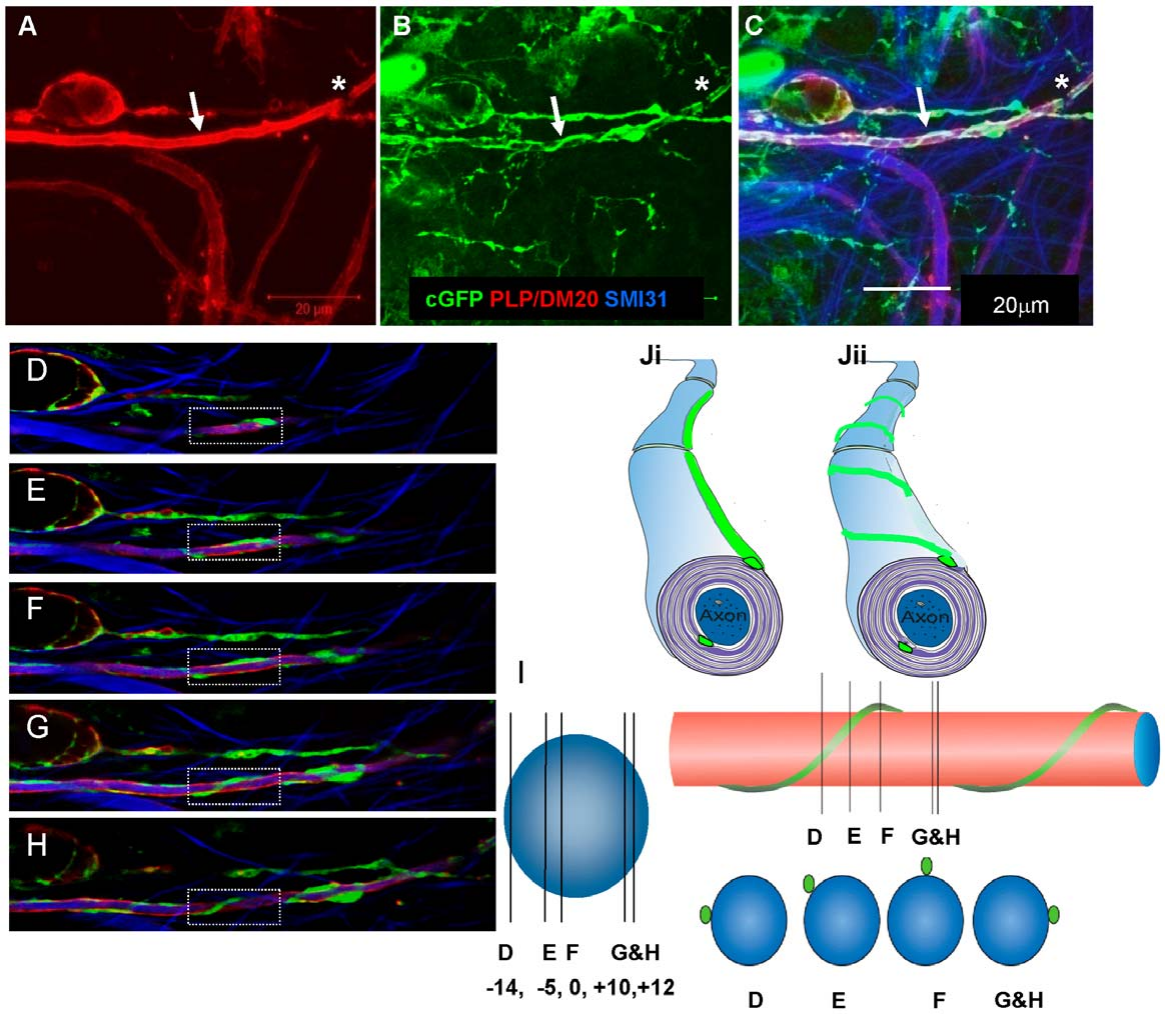
Evidence that oligodendrocytes form spiral processes around neurites. **A–C**) Confocal image acquired using a Zeiss 710, (63×, 1.4NA) with maximum projection of a z stack, with step interval of 0.06 µm of a wild type culture with exogenous added cyto-GFP labelled neurospheres, immunolabelled on 27 DIV using anti-GFP and anti-PLP/DM20. Cyto-GFP strands were visualised to spiral around an neurite on top of intense PLP/DM20 immunoreactivity. **D–H**) To confirm that the process is contiguous around the neurite, we took serial confocal images of a z stack with a step size of 0.24 µm in depth, of a cyto-GFP (green) strand spirally looping around a myelinated axon (MBP, red) **I**) The pattern of axonal wrapping is summarised in the adjacent schematic where D–H represent the images in Fig. 5D–H) visualised in cross section and longitudinally. **Ji–ii**) Illustrates a schematic of the myelinated fibre and the cyto-GFP immunoreactivity. **Ji**) Illustrates the typical schematic of a myelinated fibre with the lateral cytoplasmic loop forming a straight line. In our images it appears that this may not be the case and that it forms a spiral over the axon, at least during wrapping. Representative images from at least 5 separate experiments.

Since it is technically demanding to follow cytoplasm-filled oligodendroglial processes looping around neurites using time-lapse imaging we immunolabelled fixed wild type myelinating cultures with exogenous cyto-GFP labelled oligodendrocytes (derived from neurospheres) and visualised them using confocal microscopy. A looping, ribbon-like pattern of cyto-GFP staining was observed frequently and is consistent with corkscrew-like spiralling of the cytoplasm-filled process around the axon, as demonstrated in z stacks (Fig. 5). Even when the presumptive myelin sheath was intensely and solidly stained with PLP/DM20, suggesting that the oligodendrocyte process had wrapped around the axon at least once, the spiral pattern of cyto-GFP-positive processes was retained (Fig. 5A–C). The spiralling of the cyto-GFP ribbon around the axon (Fig. 5D–H) was confirmed in confocal images in which individual z steps at various depths through the image were made, as illustrated in a schematic (Fig. 5I).

### *In vitro* time-lapse imaging of assembly of myelin-like membrane using farns-GFP labelled neurospheres in *shiverer* cultures

**Figure 6:**
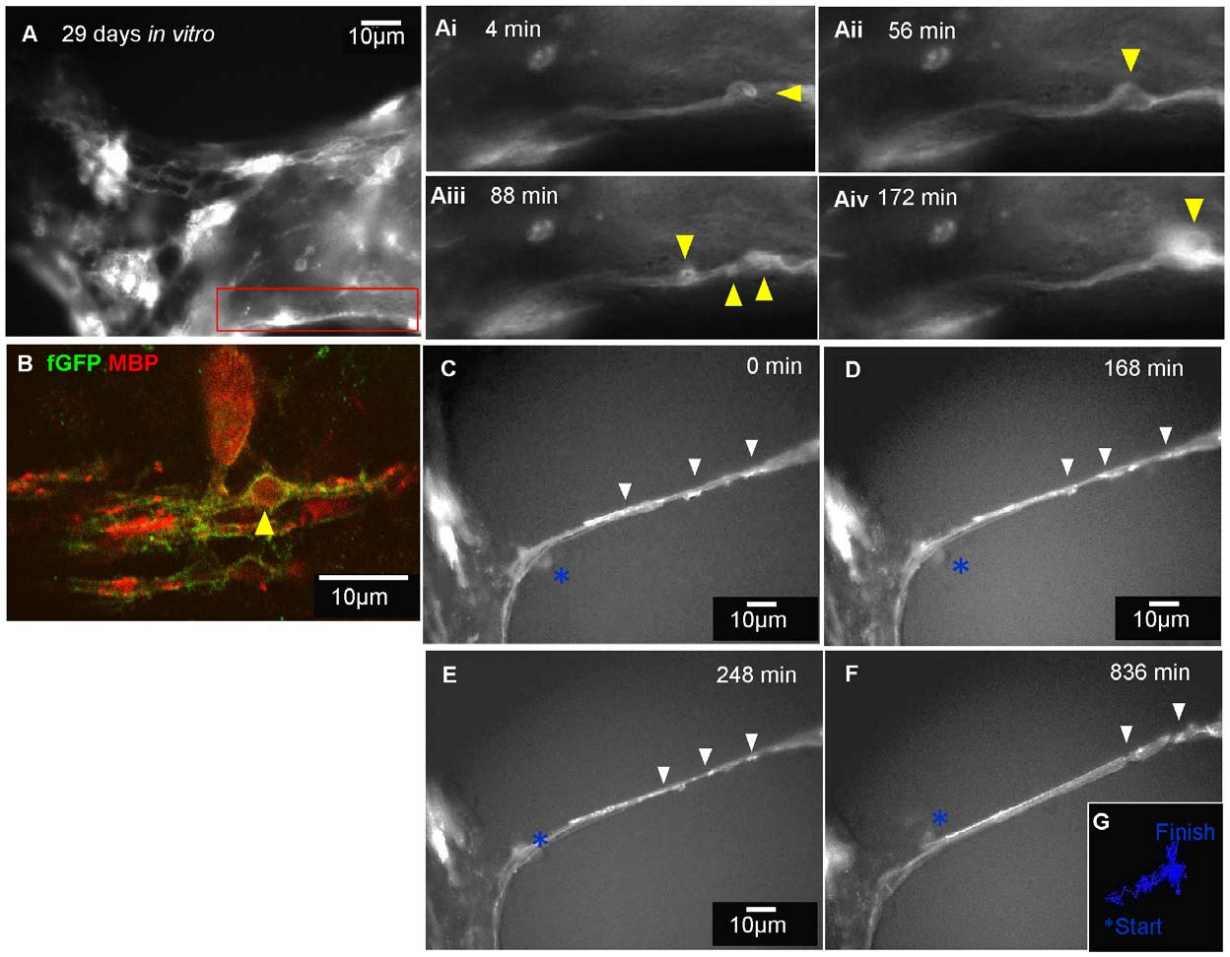
Time-lapse imaging of the putative assembly of myelin membrane. **A**) Neurospheres expressing farns-GFP were added to *shiverer* myelinating cultures on DIV 19 and time-lapse imaging (Nikon TE2000) was performed on 29 DIV, over 24 hr with 4 min time intervals. **Ai–iv**) Magnified view of the inset in **A** illustrates a farns-GFP process looping around a presumptive neurite and forming a membranous protrusions or ‘bubble’ (yellow arrow head). This membrane bubble appears to moves along the neurite over time. See Video S3. **B**) The cells from the Petri dish imaged with confocal microscopy were immunostained with anti-GFP and anti-MBP to confirm differentiation of cyto-GFP labelled oligodendrocytes.**C–F**) Time-lapse sequence of the same culture for a period of 30 hours, with 3 min time interval, on 24 DIV revealed membrane cuffs (arrowheads) extending and joining up over a neurite. After about 13 hours, the farns-GFP-positive cuffs were observed to form a single, united thick membrane sheath over a neurite. **G**) Manual tracking of the pathway of a weakly GFP-positive cell which was possibly associated with the membranous fragments. See Video S4. Representative images of membranous bubbles from at least 4 separate videos and membrane cuffs seen in 1 video but in at least 3 static images.

To visualise the oligodendrocyte membrane during myelination, farns-GFP-labelled neurospheres were added to *shiverer* myelinated cultures after 19 DIV. Time-lapse imaging was performed on DIV 29. Farns-GFP-positive cells formed a large network of branching processes (Fig. 6A). During the time course studied, waves of membrane were occasionally seen that moved along the length of the neuritic processes (Video S3). The initial membranous wave appeared to twist in relation to the orientation of the axon (arrowheads, Fig. 6Ai–Aiii) and later extended along the neurite to form a continuous sheath. A second membranous wave occurred at the same starting point (arrowhead, Fig. 6Aiii) moving progressively along the neurite until another one formed (arrowhead, Fig. 6Aiv). In less than 3 hours three membrane waves that resembled “bubbles” were generated and seemed to wind around the neurites, suggesting a possible mechanism of membrane deposition during the process of myelination. Immunostaining with anti-MBP confirmed that farns-GFP-positive neurospheres differentiated into cells of the oligodendroglial lineage (Fig. 6B). Immunostaining also revealed that large spherical extensions of the farns-GFP-labelled membrane, resembling the ‘bubble’ in Fig. 6Ai–Aiv, contained MBP.

In another time-lapse sequence taken from the same culture, farns-GFP membrane cuffs were observed (arrowheads, Fig. 6C, Video S4) over a presumptive axon. During the time-lapse acquisition these membrane cuffs appeared to extend and, after approximately 2 hours, joined together (Fig. 6D–E). At around 13 hours the GFP labelled membrane thickened to form a more continuous sheath (Fig. 6F). Manual tracking of the pathway of a weakly labelled GFP-positive cell, which was possibly associated with the membranous cuffs, suggests the oligodendrocyte-like cell is motile during this process (Fig. 6G).

**Figure 7:**
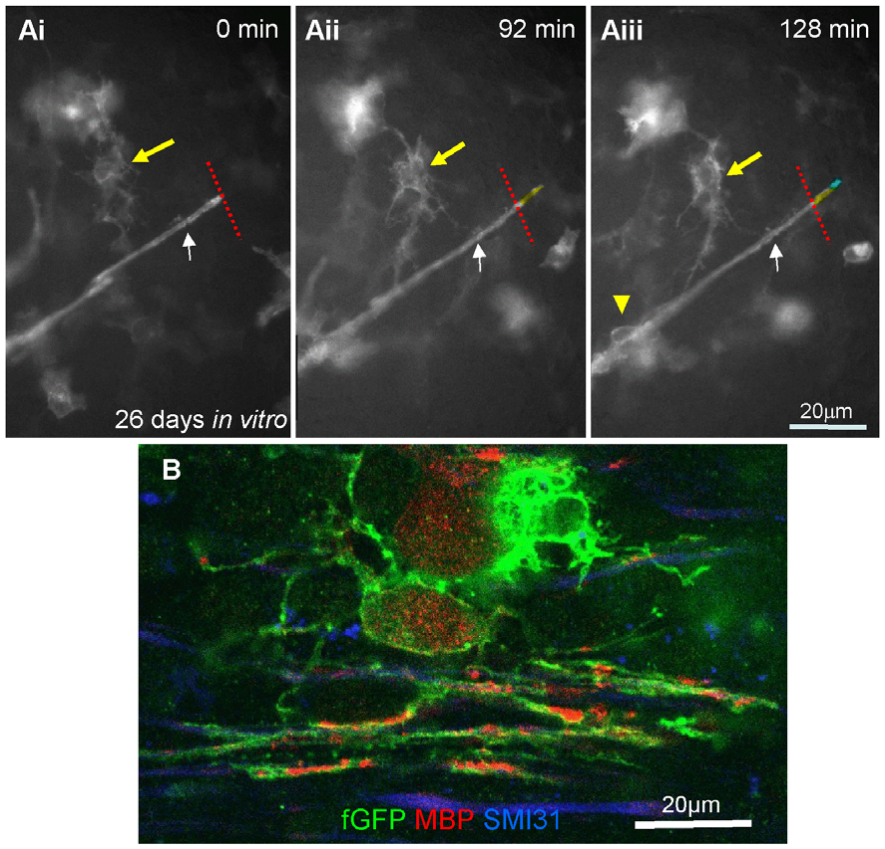
Time-lapse imaging of the elongation of a putative farns-GFP labelled myelin-like sheath. Neurospheres expressing farns-GFP were added to *shiverer* myelinating cultures on 19 DIV and time-lapse imaging (Nikon TE2000) performed on 26 DIV, for a period of 14 hr with 4 min time interval. **Ai–iii**) A farns-GFP process increases in length by 10 µm, over the time course. Membranous protrusions were seen (yellow arrowhead) in addition to the cell body of the farns-GFP labelled oligodendrocyte-like cell (yellow arrow). **B**) MBP staining of cells in the Petri dish using a Zeiss 710 (×63, 1.4NA) after imaging confirms that farns-GFP expressing cells belong to the oligodendroglial lineage. See Video S5. Representative images from at least 5 separate experiments.

Time-lapse imaging of *shiverer* cultures on day 26, at a time when the exogenous cells have formed many myelinated fibres, illustrated a farns-GFP-labelled myelin-like sheath extending longitudinally (Fig. 7Ai–iii, Video S5). The distance between the point at which the oligodendrocyte process merges with the sheath (white arrow) and the lateral edge of the sheath, increased by approximately 10 µm, i.e. ∼1/3 of its initial length, over a period of 128 minutes (see yellow and blue lines). Cultures immunolabelled after time-lapse imaging for MBP, GFP and SMI-31 provided evidence that these farns-GFP sheath-like structures are likely to represent myelin or myelin-like sheaths around *shiverer* axons (Fig. 7B).

### Myelination by transplanted GFP positive neurospheres in the spinal cord of *shiverer* mice

**Figure 8:**
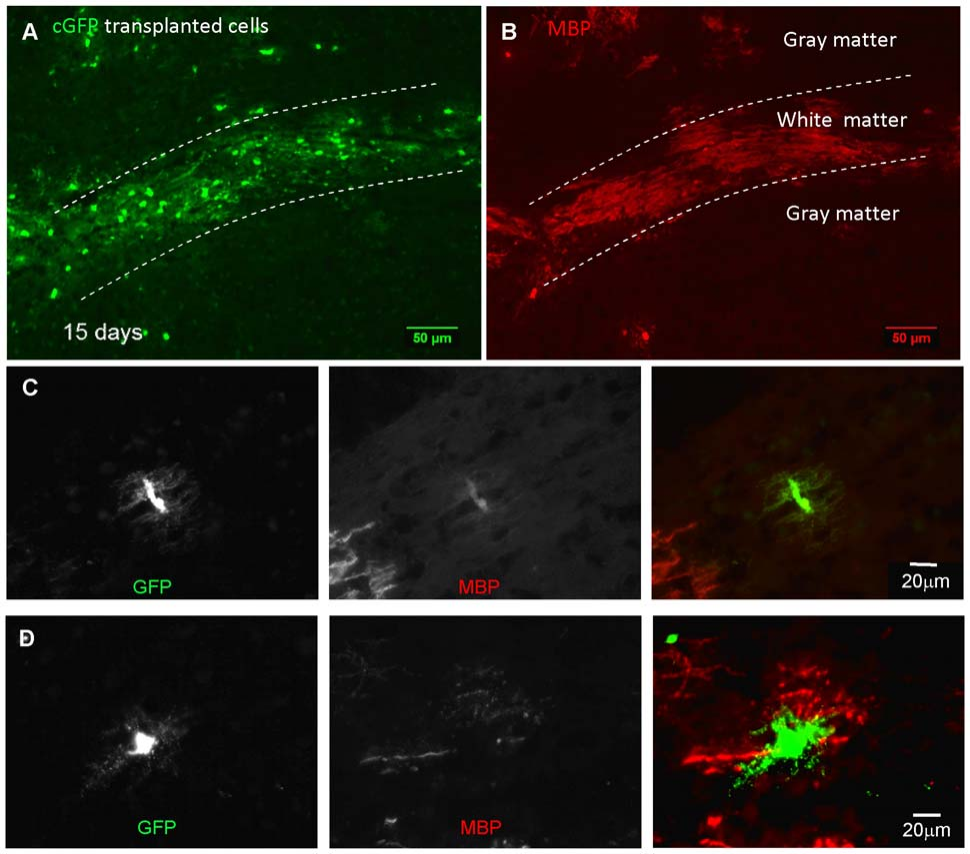
Immunohistochemistry of transplanted neurospheres demonstrate that cyto-GFP labelled cells form early and mature myelinating oligodendrocytes. Cyto-GFP-expressing neurospheres were transplanted into a *shiverer* mouse 3, 7 or 15 days post-transplantation, and 10 µm thick frozen sections were cut and immunolabelled with antibodies to GFP and MBP. Low magnification image of a dorso-vental section of spinal cord, 15 days post-transplantation showing GFP (**A**) and MBP (**B**) immunostaining. Transplanted cells were located in both grey and white matter (dorsal columns are delineated by the dotted lines) and expressed MBP-positive myelin sheaths. **C**) A pre-myelinating cell in which multiple fine GFP positive processes emanate from a central cell body. The soma is also lightly stained with MBP, confirming the identity of the cell as that of the oligodendroglial lineage. MBP-positive myelin sheaths, belonging to a second cell are seen in the bottom left hand corner of the images. **D**) An early myelinating cell in which short MBP-positive profiles are present at the periphery of the GFP-positive soma. All images were obtained using epifluorescence Olympus microscope (FV10 ASW). Representative images from at least 30 separate experiments.

To visualise oligodendrocytes *in vivo*, we transplanted neurospheres expressing cyto-GFP or farns-GFP into *shiverer* spinal cords. This method was chosen because transplanted cells become spatially dispersed, facilitating visualisation of individual cells. Before commencing live imaging of transplanted cords, we verified, using perfusion fixed tissue and immunohistochemistry, that cyto-GFP expression could be used to visualise and identify oligodendroglia. Low power images of an MBP and cyto-GFP stained dorso-ventral section of a transplanted cord 15 days after transplantation, shows co-localisation of cyto-GFP and MBP in both the grey and white matter of the dorsal spinal cord (Fig. 8A,B). At 7 and 14 days post transplantation, cells resembling pre- or myelinating oligodendroglia could be identified. Premyelinating cells were characterised by a small cell body from which a multitude of fine green processes emanated in all directions (Fig. 8C). Light staining of the soma of such cells with an antibody to MBP confirmed that they belonged to the oligodendroglial lineage. Early myelinating cells were characterised by the presence of short MBP-positive profiles at the periphery of the soma (Fig. 8D).

### Confocal images of cyto-GFP expressing cells transplanted into the s*hiverer* spinal cord revealed GFP-spirals around axons

**Figure 9:**
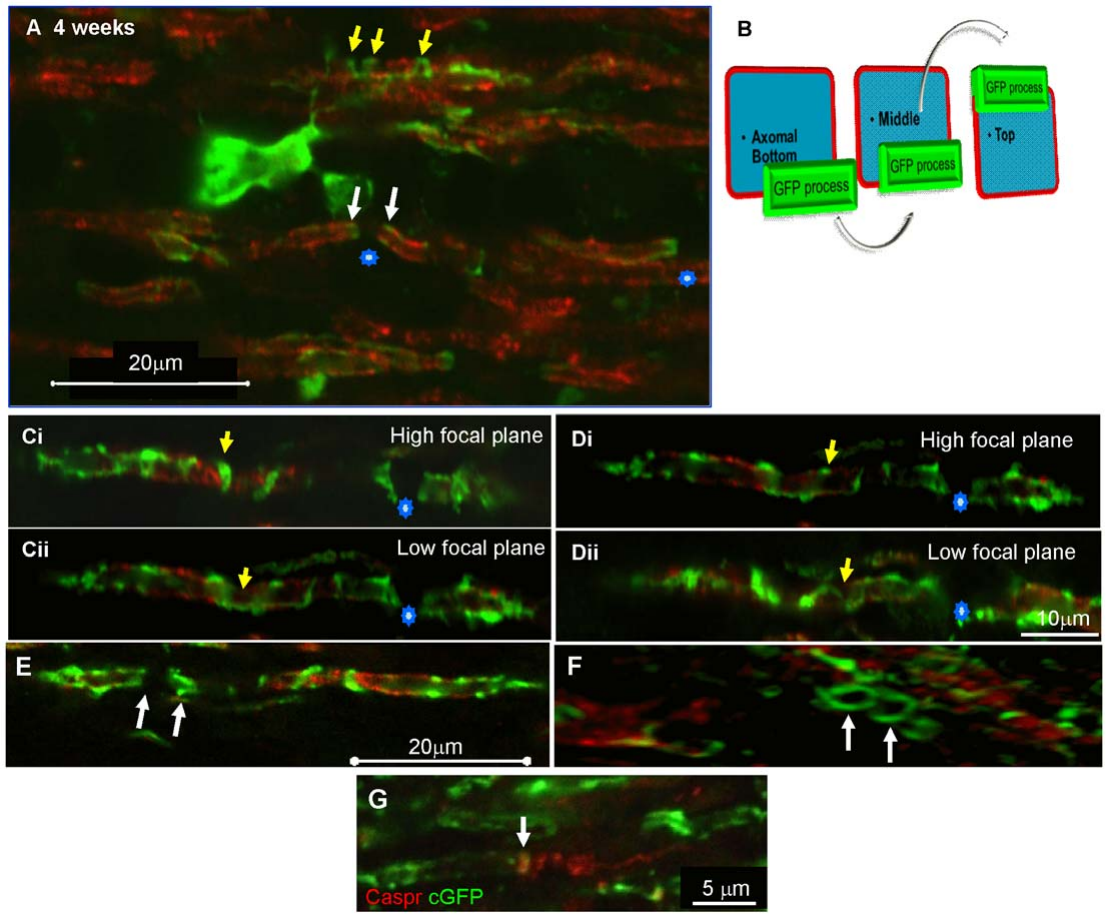
Confocal images of transplanted cyto-GFP expressing cells in the s*hiverer* spinal cord. Four weeks after transplantation of cyto-GFP expressing neurospheres, fixed sections of the *shiverer* spinal cord were immunolabelled for MBP (red) and GFP (green). **A**) A cyto-GFP labelled cell appears to extend spirals of cytoplasm around an MBP-positive myelin-like sheath (yellow arrows). Below the cell body, cyto-GFP is seen at the lateral edges (in relation to the long axis of the sheaths, white arrow) of adjacent sheaths and probably represents the cytoplasm filled paranodal loops on either side of the node of Ranvier (asterisks). **B**) Schematic of visualisation of the sections in C and D. **Ci–ii and Di–ii**) Spiral of GFP cytoplasm was followed by focussing up and down through the plane of view where they crossed up, traversed the axonal surface, then crossed down again representing the looping as shown in the schematic in B. **E**–**G**) 3D reconstruction of cyto-GFP structures (**E**), illustrates cyto-GFP either side of a space typical of a node of Ranvier (white arrows). **F**) is a tilted perspective of E) and shows the cyto-GFP form complete rings (white arrows representing the same position in E), consistent with the morphology of paranodal loops. **I**) Asymmetric caspr positive structures in association with cyto-GFP, at either side of a heminode. On the left, caspr forms a single vertical line and co-localises with cyto-GFP from the myelinating cell. On the right, caspr appears like a loose coil, consistent with its pattern of expression in non-myelinated axons. All images were acquired using an Olympus FV1000 confocal microscope (×60, 1.35NA). Representative images from at least 10 separate experiments.

Immunostaining of sections of fixed spinal cord tissue from mice transplanted with cyto-GFP-positive neurospheres demonstrated that the myelinating cells adopted a similar pattern of cyto-GFP and MBP localisation to that observed *in vitro*. A single Z slice-image of an MBP positive myelin-like sheath is illustrated in Fig. 9A. In places, cyto-GFP traversed the sheath (arrow Fig. 9A) at approximately 45° to its transverse axis, consistent with corkscrew-like spiral wrapping. By focussing up and down through the plane of view (illustrated schematically in Fig. 9B) it was possible to see the cyto-GFP-positive cytoplasmic ribbon form spirals around the axon (Fig. 9Ci–ii and Di–ii, yellow arrows). Frequently we observed cyto-GFP expression radially (in relation to the long axis of MBP-positive sheaths) on either side of a presumptive node of Ranvier (asterisks Fig. 9A), presumably representing cyto-GFP in the paranodal loops. Similar radial lines appeared as ring-like structures when images were reconstructed in 3-D (Fig. 9E,F). Co-localisation with axonal Caspr confirmed that this cyto-GFP expression occurred at paranodes (arrow, Fig. 9G,I). In some cases Caspr staining resembled that of a loosely coiled spring (right side of hemi-node, Fig. 9G), as described previously for unmyelinated axons [30]. These observations support our conclusion that cyto-GFP expression in transplanted cells permits the visualisation and identification of cytoplasmic portions of pre- and myelinating oligodendroglia.

### *Ex vivo* imaging of cyto-GFP and farns-GFP labelled transplanted cells in the spinal cord using two-photon microscopy

**Figure 10:**
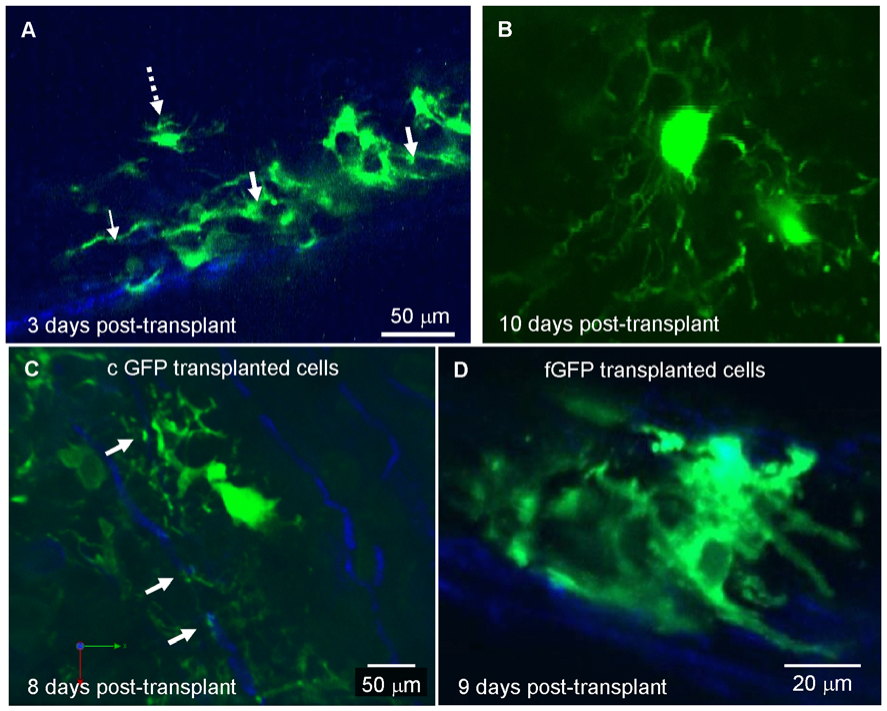
*Ex vivo* imaging of transplanted cyto-GFP and farns-GFP labelled neurospheres into the *shiverer and Thy1*-CFP*shi/shi mouse demonstrate extension of cells processes. **A**) Using multiphoton microscopy the morphology of the cyto-GFP-transplanted cells in the fixed sections of the spinal cord parenchyma was first visualised in serial images with 0.1 µm step size. Second harmonic generation (SHG) blue signal was probably generated by collagen. See Video S6. **B**) Image acquired using a Zeiss 7 MP of a cyto-GFP positive cell 10 days after transplantation into the non-fixed *ex vivo* spinal cord that resembles a typical process bearing oligodendrocyte. **C**) Eight days post-transplantation cytoplasmic GFP-positive cells were seen to extend processes making contacts with the axons at several points (white arrows). **D**) Nine days post-transplantation farnesylated GFP-labelled cells were imaged to align with the CFP-labelled axons extending thick dense processes (white arrow). B and D acquired using Zeiss 7 MP microscope (×20, 0.95NA). A and C acquired using the LaVision BioTec TRIM scope, (20×, 0.95NA). Representative images from at least 3 separate experiments.

To visualise the dynamic behaviour of transplanted cyto-GFP and farns-GFP expressing cells in *shiverer* spinal cord, we used two-photon microscopy of an ∼15 mm longitudinal portion of the *ex vivo* spinal cord, encompassing the transplant site. The *ex-vivo* cords were maintained at 37°C for up to 5 hours during imaging. In the transplanted cord cyto-GFP expressing cells were usually found as aggregates, with cell density decreasing towards the periphery of the aggregate, as illustrated in a fixed whole spinal cord (Fig. 10A, Video S6). In the centre of the aggregate, the morphology of individual cells could not be discerned. Towards the periphery of the aggregate, individual cells resembling oligodendroglia were observed (broken arrow). A single z-section through such a cell is shown in Fig. 10B. We compared the morphology of cyto-GFP and farns-GFP labelled cells transplanted in a *shiverer* mouse expressing CFP under the *Thy-1* promoter to co-visualise cells and axons. Imaging of *ex vivo* cord 7 and 8 days post-transplantation, revealed both types of GFP labelled cells extending processes associated with the CFP-positive axons. Cyto-GFP cells showed a different morphology in their association with the CFP-positive axons when compared to farns-GFP transplanted cells and could be seen to extend processes which made contacts with an axon at several points (arrows, Fig. 10C). On the other hand, farns-GFP-labelled cells, which were imaged approximately at the same time post-transplantation, appeared to align with the CFP-labelled axons with thicker processes (Fig. 10D). This reflects the differential localisation of the GFP in the cytoplasm or membrane.

### *Ex vivo* time-lapse imaging of cyto-GFP and farns-GFP labeled transplanted neurospheres into the Thy1-CFP*shi/shi mouse

**Figure 11:**
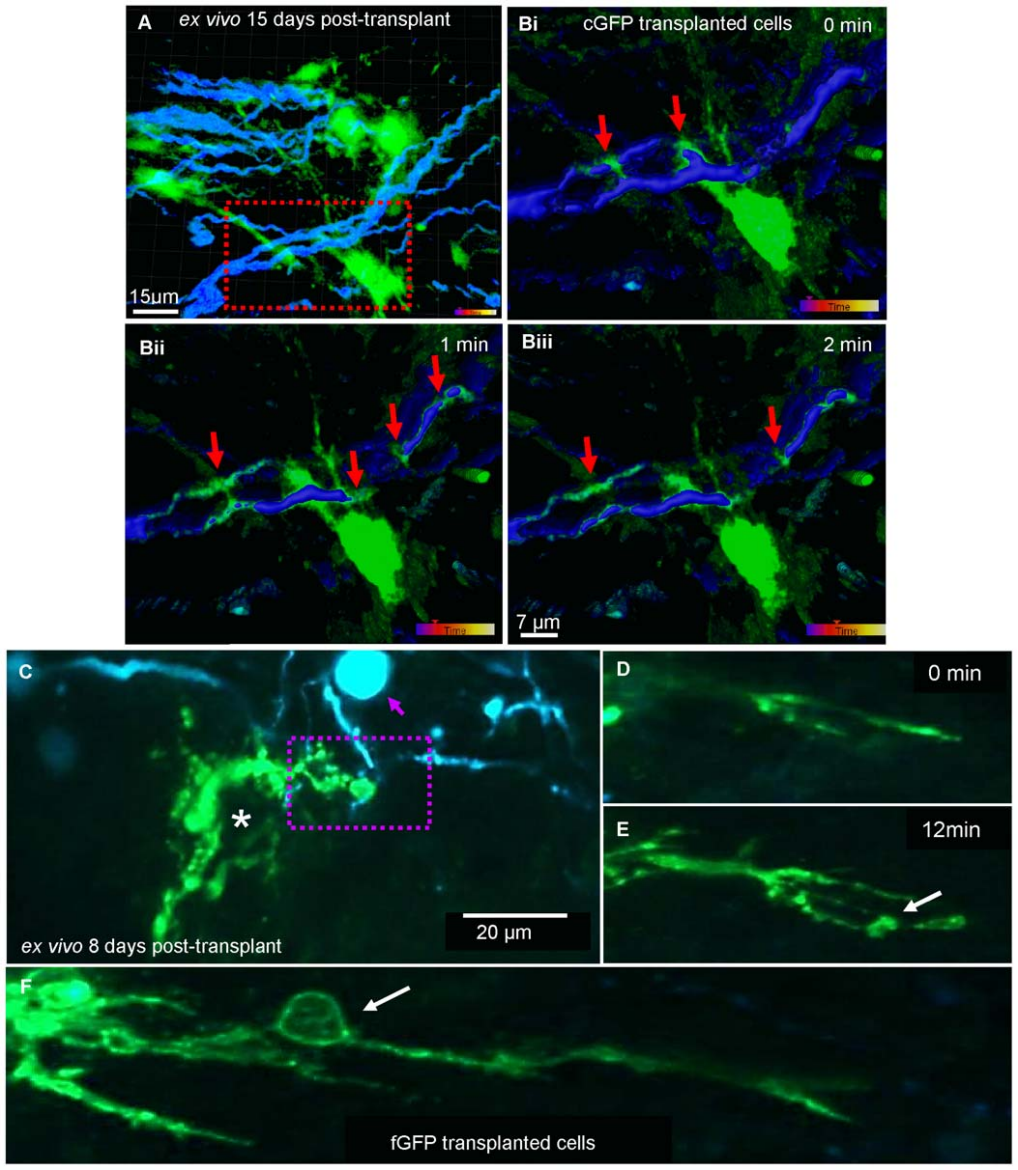
*Ex vivo* imaging of axon-glial interactions after transplantation of cyto-GFP and farns-GFP labelled cells into a *Thy1*-CFP*shi/shi mouse. **A**) Cyto-GFP-expressing neurospheres were transplanted into a *shiverer* mouse expressing CFP under the *Thy1* promoter, *Thy1*-CFP*shi/shi. Z stacks at 1 µm intervals were acquired with the multiphoton microscopy of an *ex vivo* spinal cord at day 15 after transplantation, and presented as a volume projection, over a time course of 20 min. Cyto-GFP-positive processes extending from a cell body can be seen to align with the CFP-positive axons. Z interval size was 1 µm. GFP and CFP were excited simultaneously at 860 nm. **Bi–iii**) Smoothed illustration after applying blend configuration in Imaris imaging software of the co-localization of GFP positive processes with CFP positive axons acquired using time-lapse microscopy. It can be seen that the GFP-positive cell process appears to extend over areas overlying axons. See Video S7. C–F) *ex vivo* imaging of farns-GFP-labelled cells and CFP-positive axons 8 days post-transplantation. (A–B acquired using the LaVision TRIM microscope, 20×. 0.95NA) **C**) farns-GFP-transplanted cells (asterisk) were imaged to extend flatten processes, making contact with the CFP-positive axons (dotted square), 8 days post-transplantation into *Thy1*-CFP*shi/shi mouse. In the same field of view, a neuronal body (purple arrow) was also imaged. **D,E**) Time-lapse imaging over a z-stack, with 1 µm step size, illustrating the generation and extension of a farns-GFP-positive cell process with the formation of new membrane bubble (arrow, see Video S8). **F**) farns-GFP processes were generally thick and formed membrane protrusions (arrow). C–F images were acquired using Zeiss 7 MP (20×, 0.95NA). Representative video (A,B) from 1 experiment but static images taken from at least 5 separate experiments. C–F illustrates representative images from at least 3 separate experiments.

Fifteen days after transplantation into Thy1-CFP*shi/shi mice, cyto-GFP labelled cells were visualised extending processes that associated and aligned with the CFP-positive axons (Fig. 11A). Time-lapse images demonstrated dynamic changes in the cyto-GFP process over time in the *ex vivo* cord (Fig. 11Bi–iii, Video S7). Analysis of the *ex vivo* cord 8 days post transplantation of farns-GFP cells revealed the extension of flattened membranous processes around the CFP-positive axons (Fig. 11C). *Ex vivo* imaging, through a z-volume, demonstrated farns-GFP-labelled cells extending many branches and forming occasional membrane protrusions (Fig. 11D,E, Video S8). In the *ex vivo* cord, farns-GFP labelled cell processes appeared thicker compared to the cell processes of the cyto-GFP labelled cells (Fig. 11F) and many farns-GFP-positive membrane protrusions were observed which were similar to those detected in the in vitro cultures.

## Discussion

Myelination is fundamentally a problem of topography: how is a single oligodendroglial process as we have observered in this study transformed into a compact myelin sheath? In this study we demonstrated using time-lapse imaging of cells *in vitro* and *ex-vivo* the dynamic behaviour of oligodendroglia-like cells as they engage with axons in order to generate myelin-like sheaths. Using cells expressing cyto-GFP or farns-GFP we were able to visualise cytoplasmic or membranous-aspects of oligodendroglia. Taken together, our results suggest that oligodendrocyte processes engage with axons by wrapping, like the threads of a screw, around axons, before extending longitudinally (in relation to the length of the axon), to generate myelin internodes.

The process of myelination is very complex and the visualisation of the way in which glial cells ensheath an axon is technically demanding, due in part to the complexity and dynamic nature of the process. However advances in microscopic technology have allowed more detailed visualisation of this process. Although molecular studies have provided information about signalling molecules that regulate the various stages of myelination [30], [32], less is known about the dynamic changes in cell morphology that occur over time. It has been reported that an individual oligodendrocyte can generate up to 60 separate myelin sheaths [2], [6] each from a single cell process spirally wrapping an axonal segment [33]–[35]. This fact precludes the possibility that the cell body could rotate around the axon in order to deposit myelin. Rather, formation of the spiralled myelin sheath must happen at the level of the cells' processes. Several theories have been put forward. For example, the “carpet crawler” model, in which a sheet-like cell process embraces an axon and then the leading edge completes one turn synchronously around the entire internodal length, before moving under the growing sheet to form a second wrap [12], [34], [36]. However, EM studies have shown that, at differenct locations along the length of a single developing internode, the number of wraps of membrane varies [2], [5], [37]–[40]. Thus, it is unlikely that myelination occurs through the synchronous wrapping of the leading edge of a single sheet-like process around the axon. For the same reason, the possibility that the sheath grows from the outer loop, and rotates around the axon as a ridgid cylinder constantly thickening radially and elongating at both ends, has also been dismissed [36].

### *In vitro* observations

Confocal imaging of myelinating cultures, after fixation, labelled with cell specific cytoplasmic and/or membrane markers, cyto-GFP, PLP/DM20 or the O4 antibody, allowed us to observe initial spirals of the oligodendrocyte membrane around neurites. At progressively later stages, gaps between spirals appeared to fill in with membrane, evetually a solid sheath-like structure was formed, that stained intensely with PLP/DM20 or MBP. Since cyto-GFP should be localised to the cytoplasm-filled portions of the oligodendrocyte and does not reflect all of the membrane, we labelled cyto-GFP labelled cells with the O4 surface myelin marker to determine if the markers colocalised. In Fig. 2 anti-GFP and the O4 antibody labelled cytoplasm-filled processes along the length of the axon which then formed a network of cyto-GFP spirals which were not always localised with-PLP/DM20. PLP/DM20 staining appeared to be more uniform while spirals of cyto-GFP were in general much narrower (see also Fig. 2C). Taking a series of stacked images of cells labelled with anti-GFP and anti-PLP/DM20 we were able to follow the cyto-GFP-filled spirals of membrane. This lead us to hypothesis that we were visualising an adaxonal or abaxonal cytoplasmic loop of the oligodendrocyte as a spiral rather the classical straight line that is usually depicted on the myelinated axon (see Schematic in Fig. 5J).

To determine if more information could be obtained on the initial interaction of oligodendrocytes and axons we performed time-lapse imaging of *shiverer* myelinating cultures to which cyto-GFP labelled neurospheres were added. Cells that were morophologically similar to oligodendrocyte precusor cells could be seen to continually extend and retract processess, forming a motile network of processes over the nerve bundles as well as rapidly moving along nerve fascicles. This suggest that the cytoplasmic spirals visualised using immunocytochemistry were initially formed by OPC-like cells moving rapidly over the surface of nerve fibres throwing out processes and forming many membranous protusions. Nonetheless, within the time frame in which imaging was carried out, it was not possible to distinguish between what may be transient explorations of axons by glial processes and what appear to be permanent or semi-permanent structures.

Time-lapse imaging of farns-GFP neurospheres added to *shiverer* myelinating cultures, demonstrated dynamic changes in the oligodendrocyte membrane over time. We visualised two distinct processes; i) In the first example of glia-axonal interactions, the glial process wraps an axon with a myelin-like sheath. The changes are very dynamic and ‘bubbles’ or protrusions of membrane were observed moving along neurites. When cultures were immunolabelled with differentiation markers at the end of the experiments we could detect MBP-positive GFP-positive membrane extrusions, suggesting they contained cytoplasmic MBP (Fig. 6 and 7). It is possible that these protrusions are generated by actomyosin based contractility which pushes the plasma membrane forward before it moves over the axon as a sheath [41]. ii) The farns-GFP myelin like-sheath expanded radially over an axon in cuffs and in some cultures an apparent myelin sheath extended longitudinally (in relation to the length of the axon) over time (Fig. 6 and 7). It has been suggested that myelin sheath extension [42] could occur (i) through synchronous lateral growth of consecutive layers of the mature sheath or (ii) through longitudinal extension of consecutive sheaths as they slip over each other in the spring-like (or ‘serpent’) model or (iii) through the addition of consecutive, increasingly wide, membrane sheets, from the outer loop. The idea that the sheath grows from the external loop has largely been ruled out [37], therefore it seems most likely that extension occurs through growth of the sheath or its developing layers (whether it be one or more layers thick radially) at its lateral edge i.e. at the paranodal loops.

EM studies of the CNS of rats during ensheathment and initial myelination showed cuffs of glial cytoplasm [2] which could represent the cytoplasmic cuffs seen in our cultures (Fig. 2 and 9). We demonstrated using a series of confocal images, that a spiral of GFP-cytoplasm appeared to be detected on top of both PLP/DM20 and MBP-positive myelin-like sheath, in a ribbon–like structure, resembling the model of Hirano and Dembitzer [36] in which they propose that myelin formation can occur from isolated cytoplasmic islands which slip over each other to form myelin internodes. Thus, it has been suggested that a structure around the axon which is shaped like the coil of a spring (“serpent” model) is formed by a narrow oligodendroglial process that, once wrapping is complete, extends longidtudinally (in relation to the axon) so that consecutive coils of the ‘spring’ slip over the one below (combination of hypotheses by [2], [4], [12], [36]. By this mechanism, different numbers of wraps could occur at different internodal locales, in line with the EM data [36]. It has also been suggested that each myelin sheath is formed as many abutting glial processes flatten and fuse together. In this manner the myelin sheath is formed in a similar manner to a patchwork quilt of plasma membrane from many different processes [39], [40]. Recently a liquid croissant model of myelination was proposed which can explain some of the features observed in our study. In this model the authors take in to account the uneven contours of the developing myelin sheath but also suggest that the myelin sheath is thicker at the middle of the myelin internode than at the edges [12]. This latter feature was not oberved in our experiments.

The data reported in this study suggest myelination involves a series of dynamic interactions that begins with the initial contact of an OPC process that moves along neurites forming spirals of cytoplasm-filled membrane from which myelin membrane “fills-out”, supporting the “serpent” model [4], [12]. Further, real time imaging suggests this membrane sheath forms cuffs which thicken, join up, and then extend along the axon. Our live imaging of this process should help to define early events in oligodendroglial-axonal interactions and ensheathment and are compatible with EM results reported in the literature. Thus our data suggest that myelination involves a combination of previously proposed mechanisms but the “serpent” model predomintes with aspects of the “patchwork quilt” model, thereby we term this the “ofiomosaic model” ( = όϕις+μωσα
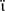
κό, Greek, meaning «snake» in its ancient Greek form and «mosaic»).

## Supporting Information

Video S1 ***In vitro*** **time-lapse imaging of OPC-like cells in** ***shiverer*** **myelinating cultures.** Cyto-GFP labelled neurospheres were added to myelinating cultures generated from *shiverer* embryos on DIV 13 and visualised on DIV 17 over 24 hours in 4 min intervals, using a Nikon TE2000 time-lapse microscope.
(AVI)

Video S2 ***In vitro*** **time-lapse imaging of fluorescently labelled cells in association with neurites.** Myelinating cultures generated from a mix of wild type/beta-actin (cyto GFP) mice were visualised using time-lapse microscopy (Nikon TE2000 (60×, 0.75NA) over 16 hr in 5 min intervals on DIV 27 after the addition of wild type neurospheres previously infected with lentivirus carrying dsRed/GFP gene and addition of cyto-GFP cells. A strongly green fluorescent cell can be seen to dynamically interact with a bundle of neurties. A red labelled cell can be seen to be engulfed by a cell resembling microglia.
(AVI)

Video S3 **Time-lapse imaging of the putative assembly of myelin membrane.** Neurospheres expressing farns-GFP were added to *shiverer* myelinating cultures on DIV 19 and time-lapse imaging (Nikon TE2000) was performed on 29 DIV, over 24 hr with 4 min time intervals. A membranous protrusions or ‘bubble’ could be detected that appears to move along the neurite over time.
(AVI)

Video S4 **Time-lapse imaging of the putative assembly of myelin membrane where the sheath is more apparent.** Time-lapse sequence of a *shiverer* myelinating culture to which farns-GFP neurospheres were added and imaged for a period of 30 hours, with 3 min time interval, on 24 DIV. The membrane cuffs extend and join up over a neurite. After about 13 hours, the farns-GFP-positive cuffs were observed to form a single, united thick membrane sheath over a neurite.
(AVI)

Video S5 **Time-lapse imaging of the elongation of a putative myelin-like sheath.** Neurospheres expressing farns-GFP were added to *shiverer* myelinating cultures on 19 DIV and time-lapse imaging (Nikon TE2000) performed on 26 DIV, for a period of 14 hr with 4 min time interval. A putative myein sheath can be seen to extend longitudinally over time.
(AVI)

Video S6 ***Ex vivo*** **imaging of transplanted cyto-GFP labelled neurospheres into the** ***shiverer*** **mouse.** The morphology of the cyto-GFP-transplanted cells in the fixed sections of the spinal cord parenchyma was visualised using the TRIM MP scope. Second harmonic generation (SHG) blue signal was probably generated by collagen.
(AVI)

Video S7 ***Ex vivo*** **imaging of transplanted farns-GFP labelled neurospheres into the** ***shiverer and Thy1*** -CFP* ***shi/shi*** **mouse demonstrate extension of cell processes.** Time-lapse video using the LaVision TRIM microscope, showing the co-localization of GFP-positive processes with CFP-positive neurites after transplantation *ex vivo*. It can be seen that the GFP-positive cell process appears to extend over areas overlying the axon.
(AVI)

Video S8 ***Ex vivo*** **imaging of transplanted farns-GFP labelled neurospheres into the** ***shiverer and Thy1*** -CFP* ***shi/shi*** **mouse.** Time-lapse video using the Zeiss 7 MP over a z-stack with 1 µm step size, showing the generation and extension of a farns-GFP-positive thick cell process with the formation of new membrane bubble.
(AVI)
